# POLICY SERIES: AGING AND HEALTH POLICY UPDATE: A VIEW FROM WASHINGTON

**DOI:** 10.1093/geroni/igad104.0558

**Published:** 2023-12-21

**Authors:** Brian Lindberg, Patricia D’Antonio

## Abstract

Leading aging and health policy advocates will present their findings and viewpoints regarding aging and health care policy during a divided Congress. Issues will include behavioral health, social isolation, health care workforce, telehealth, hospice policy, elder justice and nursing home care, home and community-based services, and Medicare and Medicaid. The group will discuss what progress has been made during the 118th Congress and what is still possible in the election year ahead.

